# Supplementary material for: Pharmacokinetics and Changes in Lipid Mediator Profiling after Consumption of Specialized Pro-Resolving Lipid-Mediator-Enriched Marine Oil in Healthy Subjects
Source: Int J Mol Sci. 2023 Nov 9;24(22):16143. doi: 10.3390/ijms242216143 (PMC10671020; doi:10.3390/ijms242216143)
Supplement: Supplementary file 1 [file ijms-24-16143-s001.zip › ijms-2689304-supplementary.pdf]

International Journal of Molecular Sciences

MDPI

Supplementary Material.

Table S1. Plasma concentrations of free fatty acids and lipid mediators' metabolomes.

	Control (water dosing)						Enriched-SPM marine oil supplementation					
	**0 hours**	**3 hours**	**6 hours**	**9 hours**	**12 hours**	**24 hours**	**0 hours**	**3 hours**	**6 hours**	**9 hours**	**12 hours**	**24 hours**
**DHA metabolome**												
DHA	51.7(45.6-67.0)	62.4(50.0-68.8)	60.0(53.0-68.7)	57.8(44.0-65.8)	55.2(47.5-61.7)	57.7(43.6-65.1)	67.7(56.2-83.0)	51.7(37.6-69.7)	54.4(39.8-62.9)	65.8(55.7-74.5)	57.5(55.7-64.4)	65.9(61.1-71.1)
14-HDHA	1.4(1.1-3.0)	1.2(0.7-2.6)	1.4(1.1-2.0)	0.9(0.6-1.8)	0.8(0.5-1.8)	1.1(0.6-3.3)	0.7(0.6-4.2)	17.4(13.9-32.3)**	32.1(13.9-39.8)**	3.9(2.2-6.2)	2.1(1.7-6.1)	2.7(1.6-4.8)
17-HDHA	0.4(0.3-0.6)	0.4(0.3-0.5)	0.5(0.5-0.6)	0.4(0.3-0.6)	0.3(0.2-0.4)	0.4(0.3-0.6)	0.4(0.4-0.5)	16.5(8.0-29.9)**	16.2(11.1-36.0)**	1.9(1.1-2.6)**	0.7(0.6-1.0)*	0.6(0.5-0.7)
RvD1	2.6(0.0-4.0)	4.0(0.0-5.1)	3.1(0.6-3.9)	3.3(0.6-5.0)	1.8(0.0-4.1)	3.2(0.7-3.9)	1.3(0.0-4.1)	4.5(1.0-7.4)	4.9(4.1-7.7)*	1.5(0.3-4.6)	2.0(0.3-2.6)	2.7(0.4-5.1)
RvD2	0.0(0.0-3.6)	1.4(0.0-5.9)	0.0(0.0-0.0)	0.9(0.0-2.5)	2.5(0.5-3.0)	0.0(0.0-2.9)	2.5(0.0-4.3)	4.2(0.8-5.3)	5.7(2.7-6.9)	2.1(0.0-3.4)	3.3(0.4-4.3)	2.7(0.2-4.5)
RvD3	1.0(0.0-1.8)	0.7(0.0-1.7)	0.0(0.0-1.2)	0.4(0.0-1.3)	0.8(0.0-1.0)	0.0(0.0-0.8)	0.7(0.0-0.9)	0.6(0.0-2.0)	1.6(0.3-2.1)	1.5(0.8-2.3)*	1.1(0.3-1.3)	0.6(0.1-1.0)
RvD4	0.0(0.0-0.0)	0.0(0.0-0.0)	0.0(0.0-0.0)	0.0(0.0-0.0)	0.0(0.0-0.0)	0.0(0.0-0.0)	0.0(0.0-0.0)	0.7(0.0-4.5)	0.0(0.0-2.6)	0.0(0.0-0.0)	0.0(0.0-0.0)	0.0(0.0-0.0)
RvD5	3.1(2.2-4.6)	4.8(3.3-17.0)	3.3(0.3-4.2)	3.6(0.6-5.8)	2.6(1.8-4.8)	3.9(2.7-4.7)	4.9(1.1-6.2)	58.3(16.6-121.1)**	23.6(15.6-43.3)**	7.9(4.7-11.6)	5.3(3.0-7.4)	5.2(2.8-7.7)
PDX	1.8(1.5-2.0)	4.6(2.2-6.6)*	2.9(0.8-3.8)	2.5(0.5-3.5)	2.2(1.7-3.7)	1.9(0.0-2.9)	1.9(1.5-3.0)	31.2(6.3-49.2)*	12.6(7.0-21.2)**	3.9(0.5-6.5)	2.9(2.2-3.7)	2.0(1.1-3.8)
PD1	1.6(0.2-3.2)	0.0(0.0-3.3)	2.3(0.0-3.6)	2.3(0.4-3.0)	3.3(0.4-4.0)	1.6(0.0-3.3)	2.3(1.7-4.1)	8.5(5.8-10.3)*	5.9(3.7-10.0)*	3.5(2.1-5.5)	1.4(0.3-3.0)	0.0(0.0-2.1)
MaR1	7.6(0.0-16.5)	9.0(7.4-24.1)	0.0(0.0-10.1)	8.8(0.0-13.1)	11.4(0.0-14.6)	2.4(0.0-19.1)	8.4(0.0-10.2)	17.1(16.0-24.2)**	3.5(0.0-19.8)	7.2(0.0-26.7)	11.8(10.1-13.4)	8.8(0.0-10.5)
MaR2	0.0(0.0-0.0)	0.4(0.0-1.6)	0.0(0.0-0.0)	0.0(0.0-1.1)	0.0(0.0-1.1)	1.1(0.0-1.9)	0.0(0.0-2.0)	39.3(23.0-62.9)**	23.1(14.7-33.1)**	3.2(1.5-6.7)*	1.8(0.4-3.4)*	1.7(0.4-3.3)*
**EPA metabolome**												
EPA	47.0(33.8-53.4)	42.9(25.2-62.9)	53.9(37.7-66.3)	44.5(27.1-51.0)	28.5(19.5-45.3)*	45.6(22.1-57.1)	37.1(25.8-55.2)	88.0(74.0-120.3)**	120.7(106.3-126.0)**	94.9(87.2-111.7)**	87.9(84.7-99.4)**	88.9(80.4-100.1)**
18-HEPE	0.2(0.2-0.3)	0.2(0.2-0.3)	0.2(0.2-0.3)	0.2(0.1-0.3)	0.2(0.2-0.3)	0.2(0.2-0.3)	0.2(0.2-0.3)	29.6(14.9-50.2)**	16.4(12.6-30.9)**	2.6(1.5-3.4)**	0.9(0.7-1.1)**	0.7(0.5-0.7)**
RvE1	0.0(0.0-0.0)	0.0(0.0-0.0)	0.0(0.0-0.0)	0.0(0.0-0.0)	0.0(0.0-0.0)	0.0(0.0-0.0)	0.0(0.0-0.0)	0.0(0.0-0.0)	0.0(0.0-0.0)	0.0(0.0-0.0)	0.0(0.0-0.0)	0.0(0.0-0.0)

**AA metabolome**												
AA	66.9(57.2-78.1)	67.8(61.4-84.1)	72.0(63.3-79.1)	68.9(62.9-76.6)	60.3(55.5-70.9)	70.7(59.0-81.9)	76.2(69.3-89.2)	73.2(65.1-75.2)	76.4(68.9-89.0)	70.3(66.9-78.0)	67.2(62.1-80.1)	76.7(70.8-84.7)
LXA_4	0.6(0.0-1.3)	1.7(0.0-2.3)	0.8(0.2-1.3)	0.8(0.1-1.3)	0.4(0.0-0.9)	0.3(0.0-1.2)	0.6(0.0-1.7)	1.7(0.2-2.8)	1.7(0.3-2.1)	1.8(0.2-2.5)	0.8(0.1-1.6)	0.8(0.0-1.4)
LXB_4	0.0(0.0-0.0)	0.0(0.0-0.0)	0.0(0.0-0.0)	0.0(0.0-0.0)	0.0(0.0-0.0)	0.0(0.0-0.0)	0.0(0.0-0.0)	0.0(0.0-0.0)	0.0(0.0-0.0)	0.0(0.0-4.7)	0.0(0.0-0.0)	0.0(0.0-0.0)
LTB4	77.0(45.6-109.6)	98.5(72.0-141.3)	90.9(38.1-141.5)	120.7(66.4-156.1)*	94.9(47.8-120.0)	142.3(75.5-185.1)*	84.9(30.8-97.0)	111.7(49.4-123.4)	88.9(43.4-125.6)	69.7(52.1-118.6)	106.7(89.4-155.1)	75.8(65.2-151.3)*
PGD_2	4.2(3.0-8.1)	6.3(2.8-9.6)	4.6(3.3-4.9)	3.9(3.2-11.1)	4.5(3.1-5.2)	4.8(3.2-6.5)	3.5(3.3-6.0)	3.7(2.3-6.2)	4.2(2.8-6.7)	3.1(2.4-4.4)	3.8(2.7-5.3)	3.3(2.6-4.9)
PGE_2	29.9(22.8-36.9)	34.1(20.2-56.1)	27.2(21.0-33.3)	28.8(22.2-39.9)	20.7(16.7-23.3)*	25.1(22.1-35.7)	14.6(11.9-15.3)	27.9(20.3-36.8)**	30.0(24.9-44.0)*	24.2(17.0-25.2)	18.6(12.5-38.6)	19.7(15.7-24.2)
$PGF_{2\alpha}$	15.3(13.9-17.9)	22.0(17.7-35.9)	18.4(14.3-20.1)	20.1(13.4-23.9)	16.7(14.9-22.1)	14.9(13.5-17.0)	16.5(13.7-22.2)	19.9(14.0-22.9)	16.8(12.3-18.0)	15.3(14.3-17.5)	17.4(15.0-25.0)	15.4(11.2-18.6)
TXB_2	362.0(212.6-482.6)	422.8(190.0-831.3)	299.8(220.8-417.5)	349.0(166.0-664.6)	272.3(164.5-387.8)	234.7(139.5-270.9)	158.1(74.3-247.6)	297.2(207.6-388.4)*	177.6(100.6-232.3)	125.4(63.6-228.3)	163.0(122.9-288.8)	154.3(78.2-177.4)
**DPA metabolome**												
DPA	50.1(42.5-62.8)	39.4(25.5-48.8)	64.0(42.4-95.7)	34.4(26.1-45.3)*	22.5(16.7-30.2)*	59.7(34.5-65.8)	42.9(36.1-51.7)	98.2(68.5-167.3)**	180.2(124.7-205.4)**	59.7(41.1-82.9)*	41.1(27.1-68.0)	58.3(45.0-82.4)*

Values are expressed as median (interquartile range) Concentrations are expressed in pg/ml, except for free fatty acids (DHA, EPA, AA, DPA) and their monohydroxylated metabolites (18-HEPE, 14-HDHA and 17-HDHA) that are expressed in ng/ml. Statistical significance, relative to time 0, within each situation as determined by by Wilcoxon Signed Rank test (*$p<0.05$;**$p<0.01$)

Table S2. Serum concentrations of free fatty acids and lipid mediators' metabolomes.

	Control (water dosing)						Enriched-SPM marine oil supplementation					
	**0 hours**	**3 hours**	**6 hours**	**9 hours**	**12 hours**	**24 hours**	**0 hours**	**3 hours**	**6 hours**	**9 hours**	**12 hours**	**24 hours**
**DHA metabolome**												
DHA	76.0(58.3-87.7)	59.7(55.6-64.4)	82.0(71.8-104.9)	70.2(61.3-93.9)	56.7(43.5-63.1)	69.0(43.9-83.2)	68.5(55.9-72.7)	55.0(52.0-61.5)	65.0(40.3-86.8)	59.7(47.5-77.2)	64.3(55.4-72.2)	64.6(57.7-82.1)
14-HDHA	109.3(76.4-134.1)	82.4(69.5-102.2)	118.9(80.3-156.0)	72.8(68.0-99.2)	56.2(51.3-82.5)*	109.4(81.4-126.6)	102.1(72.8-137.1)	175.2(123.2-209.1)	219.7(180.7-268.7)**	103.1(83.5-120.0)	102.6(81.2-126.1)	126.0(90.3-150.9)
17-HDHA	16.4(9.9-21.2)	10.4(9.2-16.6)	17.9(10.1-22.0)	11.9(8.0-14.8)	8.3(7.2-11.0)**	16.4(13.5-18.9)	13.3(10.4-27.0)	44.7(26.3-58.3)	60.5(46.4-112.6)**	16.5(11.9-20.0)	17.0(9.7-23.9)	20.2(13.7-24.0)
RvD1	0.0(0.0-0.0)	0.0(0.0-0.0)	0.0(0.0-0.0)	0.0(0.0-0.0)	0.0(0.0-0.0)	0.0(0.0-0.0)	0.0(0.0-0.0)	0.0(0.0-0.0)	0.0(0.0-3.6)	0.0(0.0-0.0)	0.0(0.0-0.0)	0.0(0.0-2.5)
RvD2	3.4(0.5-4.4)	2.9(1.8-3.9)	2.0(0.0-4.2)	0.0(0.0-2.2)	0.8(0.0-2.6)	0.0(0.0-0.9)	0.0(0.0-2.9)	0.0(0.0-1.0)	3.2(0.0-4.3)	3.5(1.9-4.6)	1.8(0.0-3.1)	2.6(0.5-3.7)
RvD3	0.0(0.0-0.0)	0.0(0.0-2.3)	0.0(0.0-1.6)	0.0(0.0-0.0)	0.0(0.0-0.0)	0.0(0.0-1.5)	0.0(0.0-0.0)	0.0(0.0-3.7)	0.0(0.0-0.0)	0.0(0.0-0.0)	0.0(0.0-1.4)	0.0(0.0-1.4)
RvD4	0.0(0.0-0.0)	0.0(0.0-0.0)	0.0(0.0-0.0)	0.0(0.0-0.0)	0.0(0.0-0.0)	0.0(0.0-0.0)	0.0(0.0-0.0)	0.0(0.0-1.9)	0.0(0.0-0.0)	0.0(0.0-0.0)	0.0(0.0-0.0)	0.0(0.0-0.0)
RvD5	48.2(27.3-77.0)	39.4(30.5-50.3)	48.2(33.0-71.9)	38.8(35.4-43.0)	33.3(26.9-61.5)	53.8(35.6-72.5)	64.5(32.3-90.7)	234.3(130.7-312.4)*	240.0(130.0-488.5)**	72.3(48.8-112.6)	60.9(47.2-111.9)	79.5(70.6-88.2)
PDX	56.9(49.1-87.1)	52.7(48.3-82.4)	58.3(47.0-122.5)	52.9(37.3-94.1)	41.9(31.9-75.0)*	79.9(39.4-104.5)	65.4(43.2-92.7)	122.2(81.4-155.3)	188.9(96.3-210.9)*	63.3(49.6-106.4)	61.7(49.2-131.6)	79.2(62.3-130.7)
PD1	7.5(5.9-11.2)	7.8(5.1-11.8)	5.1(2.4-9.8)	5.8(2.5-8.9)	3.7(2.2-6.3)*	5.6(0.7-7.9)	8.3(6.2-10.3)	13.3(9.3-19.7)**	12.8(5.9-24.3)	9.0(7.3-11.6)	6.1(4.9-12.5)	6.4(5.6-10.7)
MaR1	7.9(0.0-12.5)	18.4(11.9-20.7)*	11.7(0.0-38.1)	18.9(8.4-31.1)*	12.9(3.1-17.4)	2.9(0.0-13.7)	13.8(3.1-16.3)	22.7(9.2-65.1)	12.5(0.0-53.4)	17.8(0.0-21.8)	19.4(10.6-33.3)	10.9(0.0-20.6)
MaR2	52.1(33.9-71.8)	36.1(34.3-70.2)	55.2(33.9-87.9)	37.1(29.3-48.5)	30.2(16.4-58.4)	55.4(33.3-79.8)	47.8(23.6-125.5)	166.9(137.8-235.1)	226.3(137.9-390.5)*	47.7(40.2-76.3)	54.9(38.0-127.2)	68.3(49.2-84.0)
**EPA metabolome**												
EPA	78.6 (62.7-87.9)	82.0(67.1-87.0)	80.6(71.4-99.4)	84.0(55.5-95.1)	64.3(54.8-79.1)	61.6(45.8-84.1)	69.3(61.0-78.1)	99.1(86.1-115.9)*	134.7(99.4-155.7)**	109.2(97.0-139.2)*	116.0(103.0-123.7)*	116.2(112.0-121.2)**
18-HEPE	3.4(2.6-6.3)	2.6(2.1-5.9)	3.8(2.6-5.3)	3.2(1.7-5.4)	2.2(1.9-4.1)*	3.4(2.6-5.0)	3.4(2.3-6.4)	37.7(20.7-58.3)*	31.6(24.9-60.5)**	9.6(8.2-13.8)	10.4(6.5-14.3)	10.1(5.7-17.5)
RvE1	0.0(0.0-0.0)	0.0(0.0-0.0)	0.0(0.0-0.0)	0.0(0.0-0.0)	0.0(0.0-9.3)	0.0(0.0-0.0)	0.0(0.0-0.0)	0.0(0.0-37.3)	0.0(0.0-0.0)	0.0(0.0-0.0)	0.0(0.0-0.0)	0.0(0.0-27.9)

**AA metabolome**												
AA	101.8(88.6-125.4)	89.2(83.9-115.7)	126.6(81.4-140.6)	102.4(83.3-134.4)	87.7(81.0-105.9)	101.0(70.9-130.4)	105.1(96.5-116.2)	100.7(83.5-118.3)	105.4(85.0-128.9)	109.1(79.7-127.7)	94.9(87.1-103.4)	99.2(88.2-108.2)
LXA_4	1.3(1.0-3.3)	0.8(0.0-2.1)	1.3(0.0-1.7)	0.4(0.0-1.4)	1.7(0.9-2.2)	1.3(0.2-2.6)	1.8(1.1-3.1)	2.2(0.4-2.7)	1.9(0.4-3.1)	0.0(0.0-1.6)*	0.5(0.0-1.2)*	2.0(0.0-2.8)
LXB_4	4.3(0.0-15.3)	0.0(0.08.2)	0.0(0.0-0.0)	0.0(0.0-0.0)	0.0(0.0-0.0)	0.0(0.0-3.3)	0.0(0.0-3.9)	0.0(0.0-0.0)	0.0(0.0-0.0)	0.0(0.0-26.2)	0.0(0.0-6.0)	2.0(0.0-7.5)
LTB4	526.7(211.3-680.6)	379.8(326.3-575.2)	391.5(268.6-598.6)	267.1(217.6-360.6)	311.2(190.2-572.1)	330.0(239.0-493.7)	406.0(245.8-471.8)	428.9(267.3-574.5)	507.8(297.9-719.2)	285.6(177.9-414.9)	401.3(226.9-541.1)	474.1(289.1-591.9)
PGD_2	101.4(67.8-160.3)	109.4(67.9-172.1)	137.4(101.4-183.8)	125.0(85.8-205.5)	120.7(88.8-159.4)	118.9(79.6-413.2)	146.2(82.5-178.1)	122.5(86.5-183.5)	90.5(62.7-139.8)*	70.5(44.8-184.4)	124.9(66.1-231.2)	86.6(71.1-145.2)
PGE_2	17108.2 (14972.2-33569.9)	23277.6 (4963.2-34259.5)	23348.0 (20979.7-31014.1)	20607.8 (6011.2-43767.4)	19912.2 (14690.2-31520.3)	16471.9 (7613.6-51855.3)	27618.9 (8779.9-37633.9)	24636.2 (8194.4-40735.9)	18231.1 (5721.1-25935.5)	15037.9 (2624.0-37589.6)	19513.7 (3637.4-35650.0)	19594.4 (2631.4-26698.8)
$PGF_{2\alpha}$	3827.7 (2754.4-4001.4)	3249.5 (1963.8-4360.4)	4545.5 (3588.5-4806.8)	4199.8 (2933.9-6610.5)	4000.6 (2765.7-5034.1)	3740.5 (2234.1-8736.8)	4377.2 (3210.0-5496.6)	3732.9 (2716.5-4897.4)	3274.1 (2613.5-4359.4)	2979.8 (2162.5-5065.5)	4011.4 (2316.6-4445.1)	3315.2 (2874.6-3957.2)
TXB_2	19806.9 (14400.2-27760.3)	27790.8 (15922.1-31180.3)	38756.6 (27045.2-47334.9)	24570.9 (19173.9-32018.1)	29234.7 (19041.8-42877.4)	32345.3 (20572.8-46941.2)	27305.0 (22935.3-40094.5)	29370.3 (21000.7-32753.6)	28810.5 (22468.0-43069.4)	26064.5 (20533.9-36773.9)	24540.2 (12355.5-30008.4)	29810.3 (19209.5-36860.6)
**DPA metabolome**												
DPA	59.0(38.4-70.1)	40.0(35.3-49.6)	72.5(55.5-78.2)	38.6(34.3-49.5)	49.4(39.7-62.8)	51.8(42.9-71.3)	51.9(47.9-61.5)	125.8(82.0-147.5)*	132.2(124.7-199.3)**	64.4(57.4-80.6)	61.3(55.7-68.3)	65.9(55.7-81.8)

Values are expressed as median (interquartile range). Concentrations are expressed in pg/ml, except for free fatty acids (DHA, EPA, AA, DPA and their monohydroxylated metabolites (18-HEPE, 14-HDHA and 17-HDHA) that are expressed in ng/ml. Statistical significance, relative to time 0, within each situation as determined by by Wilcoxon Signed Rank test (*$p<0.05$;**$p<0.01$).
